# Single Amino Acid Changes Impact the Ability of *Drosophila melanogaster* Cecropins to Inhibit Growth of *Providencia* Pathogens

**DOI:** 10.1021/acsomega.4c07262

**Published:** 2025-02-05

**Authors:** Marla J. Forfar, Christopher R. Feudale, Lauren E. Shaffer, Grace M. Ginder, Marion E. Duval, Michelle Vovsha, Quinn B. Smith, Moria C. Chambers, Sarah J. Smith

**Affiliations:** † Department of Chemistry, Bucknell University, 1 Dent Dr., Lewisburg, Pennsylvania 17837, United States; ‡ Program in Cell Biology and Biochemistry, Bucknell University, 1 Dent Dr., Lewisburg, Pennsylvania 17837, United States; § Department of Biology, Bucknell University, 1 Dent Dr., Lewisburg, Pennsylvania 17837, United States; ∥ Program in Neuroscience, Bucknell University, 1 Dent Dr., Lewisburg, Pennsylvania 17837, United States

## Abstract

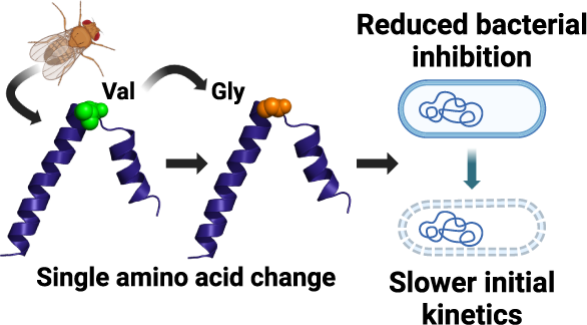

As antibiotic-resistant bacteria spread worldwide, the need to develop novel antimicrobial agents is urgent. One rich source of potential antimicrobials is the insect immune system, as insects produce a wide range of antimicrobial peptides (AMPs) with diverse sequences and structures. Insects also encounter many bacterial pathogens, some of which are closely related to pathogens of clinical relevance. However, despite interest in AMPs as therapeutics, the relationships between the amino acid sequence, biophysical properties, antimicrobial activity, and specificity are still not generalizable. To improve our understanding of these relationships, we assessed how single amino acid changes in cecropin AMPs produced by the fruit fly, *Drosophila melanogaster*, impact both their structure and their ability to inhibit the growth of *Providencia* species isolated from wild-caught *D. melanogaster*. These pathogens are of particular interest as they have a range of virulence in fruit flies, and work *in vivo* suggests that differences in virulence could be partially attributable to differential susceptibility to AMPs. *D. melanogaster* cecropins are 40 amino acids long but vary at only 5 residues with largely conservative changes. We found that these changes could impact inhibitory concentrations by up to 8-fold against *Providencia* species. Our investigation focused on a single amino acid position due to the importance of a flexible “hinge” in cecropin function. We found that altering the identity of this amino acid alone greatly impacted antimicrobial activity, changing bacterial susceptibility up to 16-fold. Generally, *Providencia* species that are less virulent *in vivo* are more susceptible to cecropin AMPs *in vitro*. We also observed differences in the kinetics of permeabilization and bacterial killing between species, suggesting that peptide-membrane interactions were differently affected by single amino acid changes and that bacteria in this genus may vary in their membrane composition.

## Introduction

The need for the development of novel classes of antibiotics has been well documented, as the world is rapidly approaching a crisis where currently available treatments are no longer effective against the spread of antibiotic-resistant bacterial strains. Recent studies estimated the cost of antibacterial resistant infections at approximately $4.6 billion in the United States alone in 2017,^1^ with 1.27 million deaths directly caused by antibiotic resistant infections worldwide in 2019,^2^ and it is expected that these numbers will continue to grow in the absence of novel classes of antibiotics.^3^ Nature contains a wealth of possible antimicrobial agents, including antimicrobial peptides (AMPs), which are produced by a wide range of organisms and have recently begun to be used as human therapeutics.^4−6^ Importantly, AMPs are believed to kill bacteria primarily by physically disrupting the cellular membrane,^7−9^ and it is expected that bacteria are slower to develop resistance to this type of mechanism.^10^ AMPs are a highly diverse group of peptides that vary in size, amino acid sequence, biochemical properties, structure, and microbial targets.^11−13^

Insects produce a variety of AMPs, and encounter a wide range of pathogens, including several that are closely related to ESKAPE pathogens (*Enterococcus faecium*, *Staphylococcus aureus*, *Klebsiella pneumoniae*, *Acinetobacter baumannii*, *Pseudomonas aeruginosa*, and *Enterobacter* species), a group of antibiotic-resistant bacteria of concern in hospitals.^14−16^ However, despite the therapeutic potential of AMPs, the relationship between the amino acid sequence, biophysical properties, and antimicrobial activity is still not generalizable.^17−19^ Here, we focus on a family of cecropin AMPs expressed in the fruit fly, *Drosophila melanogaster*. Cecropins are approximately 40 amino acid long peptides that are primarily α-helical and do not contain cysteine bonds.^20^ They consist of two α**-**helices connected by a flexible hinge region; the longer N-terminal helix is basic and positively charged, while the shorter C-terminal helix is hydrophobic.^21^ Upon infection, along with other AMPs, cecropins are secreted into the hemolymph of flies at concentrations estimated to be between 25–100 μM.^22^*D. melanogaster* expresses 4 distinct cecropin peptides, referred to here as CecA1, CecA2, CecB, and CecC. Upon removal of the signaling sequence prior to secretion, CecA1 and CecA2 have identical sequences, so we treated them as a single peptide, CecA. Similar to other cecropins that have been structurally characterized, all three of the cecropins from *D. melanogaster* have distinct hydrophobic and hydrophilic regions with primarily cationic amino acids.^23^

There are very few differences in the sequences of the three native cecropins, and those that are present are conservative mutations, maintaining a similar size, charge, and hydrophobicity between sequences (Table 1). All peptides were synthesized with a C-terminal amide group, as this is a postsynthetic modification that is found in the native peptide sequence and increases antibacterial activity.^24^

**Table 1:**
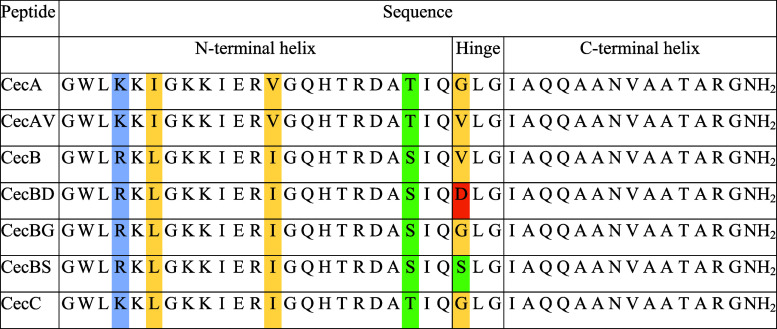
Sequences of Cecropins Used in This Work

CecA, CecB, and CecC are naturally synthesized by *D. melanogaster*, with amino acid differences between the peptides highlighted. Basic residues are shown in blue, acidic residues are shown in red, hydrophobic residues (and glycine) are shown in yellow, and polar uncharged residues are shown in green. CecAV, CecBD, CecBG, and CecBS are cecropins with single point mutations to test the importance of the identity of the amino acid at position 24. The predicted *N*- and C-terminal helices are shown, along with the flexible hinge region.

The antibacterial properties of cecropins from a variety of insect sources has been well established, and few studies have examined differences in the function of cecropins from the same family.^25^ There is some evidence to suggest that different cecropins from the same organism display different antibacterial properties.^19,26^ In the case of *D. melanogaster*, these peptides have been considered as a class, with no investigation into whether small changes in the amino acid sequence will change the antimicrobial activity of the peptides.^22,27,28^ We set out to determine whether we could observe differences in the bacterial specificity or activity of these AMPs. We hypothesized that a single amino acid change at position 24 in the native peptide sequences, in the hinge region, could be responsible for our observed large differences in the inhibition of bacterial growth, as the hinge region has previously been shown to be essential in determining antibiotic activity.^29,30^ CecB contains a Val residue at position 24, while CecA and CecC contain Gly residues at position 24, leading us to posit that the flexibility in the hinge region may differ between peptides, but few studies have directly measured the impact of changing amino acids in the hinge of native cecropins on antibacterial properties. We therefore made additional peptide variants to test the impact of changing a single amino acid within the hinge segment. CecAV, containing a G24V mutation, and CecBG, containing a V24G mutation, were synthesized to test whether we could enhance or impede antibacterial activity by altering between the two naturally found amino acid residues at position 24. CecBD and CecBS were synthesized to further test the importance of that position within the hinge region, as both mutations more drastically changed the properties of the amino acid side chain. We found that single amino acid changes at position 24 led to large variations in antibiotic activity observed by MIC and permeation results, demonstrating a potent structure–function relationship that can be exploited for altering antibacterial activity.

In our studies, we focused on testing five *Providencia* species that were isolated from field-caught *D. melanogaster*.^31^ The bacteria from this genus were chosen due to the fact that *Providencia* species have a range of virulence in *D. melanogaster*, and are bacterial strains that we expect would naturally promote the secretion of cecropins.^32,33^ Importantly, we hypothesized that the native cecropins may display different antimicrobial effectiveness depending on the bacterial species, and that differential resistance to AMPs may be partly responsible for the breadth of virulence in this genus. *Providencia burhodogranarieaD* is the mildest strain and does not cause significant mortality in *D. melanogaster* above controls.^32^*P. rettgeri* and *P. burhodogranarieaB* cause moderate levels of mortality, while *P. sneebia* and *P. alcalifaciens* are highly virulent.^32^ We predict that those that are the most virulent are the most resistant to the antimicrobial effects of cecropins, while the mildest pathogens might be the most susceptible. Supporting this hypothesis, when flies are unable to express any cecropins in addition to missing other AMPs, the flies are more susceptible to *P. burhodogranariea* but not *P. rettgeri*, although the other *Providencia* species have not been tested.^27^ However, we anticipated that *P. sneebia* might actually be susceptible to cecropins as it does not induce the *D. melanogaster* immune system during infection, and the lack of expression of AMPs by the host might lead to its high virulence.^32^ Here, we find that small sequence changes in cecropins impact the antibacterial activity with minimal impact on structure and that antimicrobial effectiveness against *Providencia* species is consistent with their relative virulence. Further, the antimicrobial effectiveness of mutant cecropin peptides is different between bacterial species, indicating that interactions between the AMPs and bacteria vary even between closely related species.

## Results and Discussion

### Sequence and Structural Analysis of *Drosophila melanogaster* Cecropins

The natively expressed cecropins from *D. melanogaster* show high sequence similarity, with amino acid changes that would not be expected to influence the structure or biophysical properties of each peptide. The entire C-terminal hydrophobic domain is identical between the three native peptides (Table 1). There are only two amino acid changes between CecA and CecC; an Ile to Leu change at position 6, and a Val to Ile change at position 13. CecB is more divergent, with an additional Lys to Arg change at position 4, a Thr to Ser change at position 21, and most notably, a Gly to Val change at position 24. However, these changes would typically be predicted to have minimal effect as they result in substitutions of the same type of amino acid (e.g., nonpolar to nonpolar).

**Figure 1:**
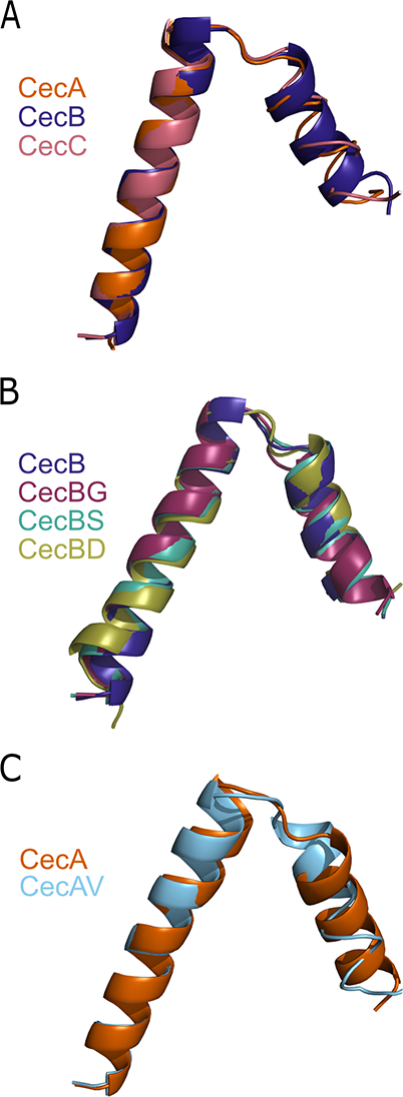
Structural modeling of the cecropin peptides. Peptides were modeled using the I-TASSER structure prediction server. The PDB models for the structures with the lowest energy were aligned. (A) Structures of the three native cecropin peptides, CecA, CecB, and CecC. (B) Structures of modified CecB peptide, with mutations to the native Val residue at position 24. (C) Structures of the modified CecA peptide.

Modeling studies were performed using the I-TASSER structure prediction server (Figure 1).^34−36^ The lowest energy structures were predicted to have a 23-amino acid helical region from positions 1 to 23, followed by an unstructured region of approximately 3 amino acids, and a second, hydrophobic, helical region. The predicted structures are similar for all of the peptides studied here. The helix-hinge-helix conformation is similar to other cecropins that have been structurally characterized and show an amphipathic N-terminal helix followed by a hydrophobic C-terminal helix.^30^ It is believed that the hydrophobic C-terminal helix can insert into the lipid membrane, and multiple cecropin peptides can associate to form a pore, disrupting membrane integrity.^20^ These hydrophobic interactions are essential for antimicrobial activity. Previous research has demonstrated the importance of the hinge region linking N-terminal cationic and C-terminal hydrophobic α-helices.^29^ It has been hypothesized that flexibility in the hinge region may be important for the ability of the peptide to span and disrupt the bacterial lipid membrane, directly impacting its antimicrobial activity.^29^ As a result of the sequence analysis and modeled structures of the cecropins, we predicted that the amino acid change most likely to affect activity between the native cecropins would be the Gly to Val change at position 24. Therefore, we chose to make additional peptides to compare the effectiveness of different amino acids in this location on the antimicrobial properties of the peptide. CecAV contained a Gly to Val mutation at position 24 compared to the native CecA sequence, and CecBG contained a Val to Gly mutation at position 24 compared to the native CecB sequence (Table 1). We also synthesized CecBS, with a Val to a polar Ser mutation at position 24, and CecBD, with a charged Asp residue at position 24. Based on I-TASSER predictions, no significant differences in the structure were expected (Figure 1B,C) due to alterations at position 24. Further, the hydropathy of each peptide was analyzed using MPEx,^37^ and only CecBD has an observable difference in hydropathy profile (Figure S1). All peptides were synthesized using standard solid-phase peptide synthesis with FMOC chemistry and purified via preparatory scale high performance liquid chromatography (HPLC). Peptide sequences were confirmed by electrospray ionization mass spectrometry (ESI-MS) and purity of >95% was verified by analytical HPLC prior to use in experiments (Figure S2). The analysis of each peptide is provided in the Supporting Information.

**Figure 2:**
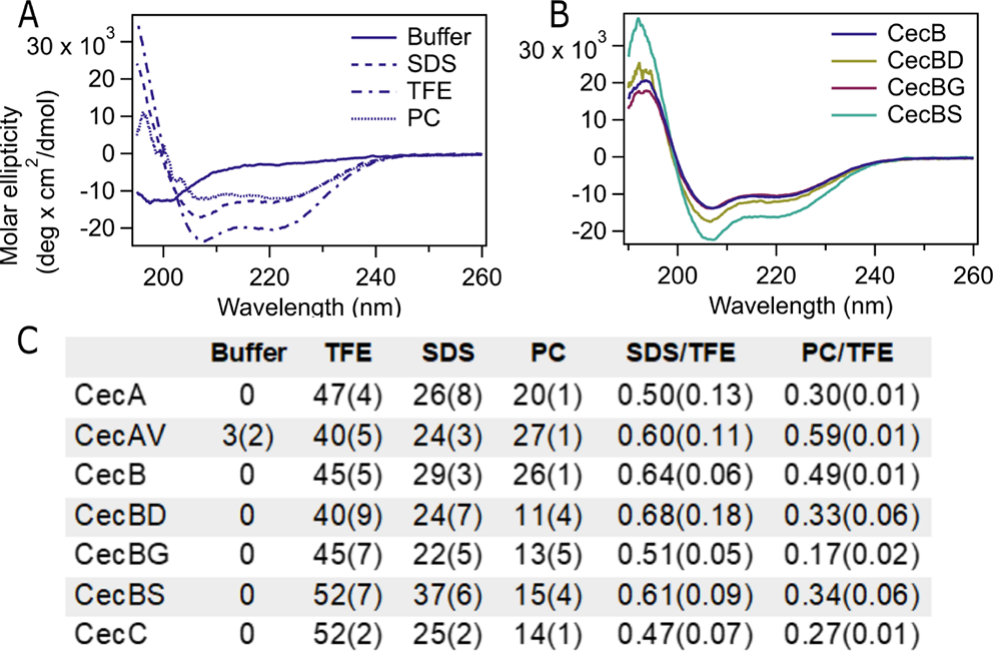
Circular dichroism spectroscopy studies of the secondary structure of native *D. melanogaster* and mutated cecropins. (A). Representative CD spectra of CecB peptide in buffer (solid line), sodium dodecyl sulfate (SDS) (dashed line), trifluoroethanol (TFE) (dashed-dotted line), and soy L-α-phosphatidylcholine (PC). All peptides shifted from a random coil structure in buffer with a single minimum at approximately 200 nm to an α-helical structure in SDS, TFE, and PC with minima at 208 and 222 nm and a maximum at 195 nm. (B). Measured CD spectra of the CecB mutant peptides compared to the native sequence. Spectra shown are averages from three separate samples. (C). Estimations of the percent helicity of each peptide were calculated using BestSel.^39^ Each peptide was measured in triplicate from separate sample preparations, the percent helicity was calculated separately, and the values were averaged. The standard deviations are in parentheses. Peptides have essentially no helical characteristic in buffer. The ratios of helicity were calculated for each trial to minimize errors in peptide concentration and then averaged.

In order to experimentally determine the secondary structure of each of our cecropin peptides, circular dichroism (CD) spectroscopy was performed. As has been demonstrated with other peptides in the cecropin family, the peptides are unfolded or in random coil structures in buffer as demonstrated by the single minimum in the CD spectrum at approximately 200 nm (Figure 2A).^38^ The percent helicity for each peptide was estimated using BestSel (Figure 2C).^39^ BestSel fitting confirmed that there was no helical contribution to the secondary structures of each of the peptides in buffer. The mechanism of action for cecropins to disrupt the cellular membrane is proposed to depend on their ability to fold into an α-helical conformation in the presence of lipid membranes.^40^ Therefore, the secondary structure of each peptide was determined in solvent conditions that mimic the membrane environment. Each peptide was diluted in a solution containing sodium dodecyl sulfate (SDS) at a concentration above the critical micelle concentration. This solution mimics the membrane environment, and SDS is negatively charged which better mimics the lipid composition of bacterial membranes. All seven cecropin peptides shifted to a more helical secondary structure, as demonstrated by the double minima located at 208 and 222 nm, which is consistent with the expected mechanism for membrane disruption by cecropins.^41^ Minimal difference in the secondary structure was observed among all peptides, the three native cecropins and four mutated sequences. However, all three peptides containing Gly at position 24 (CecA, CecBG, and CecC) had among the lowest calculated percent helicities in SDS (Figure 2). This is likely due to the increased flexibility of the peptide backbone when Gly is present at position 24, and expected as Gly is generally unfavorable to be present in helical structures. Trifluoroethanol (TFE) is commonly used to stabilize secondary structures in peptides and proteins.^42^ As expected, we also observed a shift to a helical conformation in a solution containing 50% TFE, and all peptides were more helical in TFE compared to SDS. We compared the ratio of helicity in SDS and TFE (Figure 2C), expecting that the maximal amount of helicity would be measured in TFE. Again, for the peptides containing Gly at position 24, the extent of helix induction was lower in SDS compared to other peptides, although CecBS was slightly more helical in SDS when compared to the native CecB peptide. We hypothesize that in the presence of TFE, the peptides are primarily in a single, extended α-helical structure, and that interactions with the SDS micelle are more likely to result in a helix-hinge-helix structure. Further, it is possible that the lower ratio of helicity in SDS compared to TFE for peptides with Gly at position 24 is due to additional disorder in the hinge region, especially as the terminal regions of both helices are identical. The secondary structure of each peptide was also measured in the presence of liposomes consisting of Soy l-α-phosphatidylcholine (PC) in order to better mimic bacterial membranes. Similar trends were observed, and the two peptides containing Val at position 24 (CecAV, CecB) had the highest measured PC/TFE helicity ratio, leading us to hypothesize that the presence of Val in the hinge region allowed for better folding upon membrane binding. Notably, comparatively less helicity was observed for both CecBD and CecBS in the presence of PC compared to SDS, indicating that less conservative mutations have a greater impact on structure in a membrane.

### In Vitro Antimicrobial Activity of Peptides against Bacteria That Infect *D. melanogaster*

Each cecropin was tested for its ability to inhibit the growth of *Providencia* bacterial species, strains that were previously isolated from *D. melanogaster*.^31^ Not only do these species exhibit a range of virulence when infecting *D. melanogaster*,^32^ but removal of all four cecropin genes *in vivo* affects survival during infection with the least virulent species, *P. burhodogranariea*.^27^*P. rettgeri*, a more virulent strain of *Providencia*, is highly lethal whether or not cecropin genes are expressed.^27^ Therefore, we hypothesized the virulence of *Providencia* species may be partially due to differing susceptibility to cecropins. Experiments were conducted at 25 °C (Table 2), which is the temperature at which *D. melanogaster* are typically housed and likely reflects the temperature of bacterial growth *in vivo* as fruit flies are poikilotherms. A standard 96-well plate microdilution assay was used to determine the minimum inhibitory concentration (MIC) for each bacterial-cecropin combination, with concentrations ranging from 1 to 64 μM cecropin (or 128 μM in select cases).^43^ Bacteria were determined to be in the log phase of growth when assays were started (Figure S3). *P. burhodogranarieaB* and *P. burhodogranarieaD* were the most susceptible to growth inhibition by all three native cecropin peptides (Table 2), which is unsurprising, given their low virulence and ability of *D. melanogaster* to sometimes clear infection by these bacteria.^32^*P. sneebia*, *P. alcalifaciens*, and *P. rettgeri* were more resistant to all three cecropins, with no growth inhibition observed against *P. alcalifaciens* in any condition. While *P. sneebia* was generally more resistant, it was susceptible to both CecB and CecC at physiologically relevant concentrations, suggesting that its high level of virulence may be partially due its poor induction of the immune system.^32^

**Table 2:** Minimum Inhibitory Concentrations (μM) for Cecropins at 25 °C

	CecA	CecAV	CecB	CecBG	CecBD	CecBS	CecC
*P. burhodogranarieaB*	8	4	2	8	>128	4	4
*P. burhodogranarieaD*	16	2	2	32	>128	16	4
*P. sneebia*	>64	32	32	128	>128	>128	64
*P. rettgeri*	>64	64	64	>64	>128	>128	>64
*P. alcalifaciens*	>64	NT	>64	NT	NT	NT	>64

Overall, CecB was the most effective at inhibiting bacterial growth of the 3 natively expressed cecropins, CecA, CecB, and CecC. CecB demonstrated the highest level of activity against *P. sneebia*, with an MIC of 32 μM and was also the only peptide to demonstrate activity in inhibiting the growth of *P. rettgeri*, with a measured MIC of 64 μM. All three native peptides inhibited growth of *P. burhodogranarieaB* and *D*, but the inhibitory concentrations for CecB were lower than for CecA and CecC. Even though the amino acid changes between these native cecropin peptides appear to be conservative, they impacted antibacterial activity, shifting the MIC by up to 8-fold for a given bacterial species.

We hypothesized that the presence of Val instead of Gly in CecB was responsible for the higher antimicrobial activity against *Providencia* bacteria. We therefore designed two mutants, CecAV and CecBG to isolate the effect of a Gly versus a Val residue at position 24. We expected the presence of Val at position 24 to increase the antibacterial effect of CecAV compared to CecA (resulting in a lower observed MIC), and the presence of Gly a position 24 to decrease the antibacterial properties of CecBG compared to CecB (resulting in a higher observed MIC). The MIC decreased for all bacteria when comparing CecAV to CecA, with the most dramatic enhancement observed in a 16-fold decrease in the MIC for *P. burhodogranarieaD*. Conversely, the MIC increased when comparing CecBG to CecB, again most dramatically in the case of *P. burhodogranarieaD,* where a 32-fold increase in MIC was observed (Table 2). Both peptides that have a Val in position 24, CecB and CecAV, were able to inhibit *P. rettgeri* with an MIC of 64 μM, but the peptides with a Gly at position 24, CecA and CecBG, had no inhibition of *P. rettgeri* at the doses tested. Together these results confirm that the identity of the amino acid at position 24 in the hinge region is important for antibacterial activity and that having a Val in this position enhances activity compared to Gly. We hypothesize that this is likely because increased flexibility in the hinge region due to Gly at position 24 is detrimental to the insertion and interruption of the bacterial membrane. Alterations to activity are also mirrored by changes in structure; peptides containing Gly at position 24 (CecA and CecBG) generally had lower helicity in the presence of SDS relative to their structures in TFE compared to their counterparts containing Val at position 24 (CecAV and CecB) (Figure 2B). This increase in helical signal may also be due to decreased flexibility in the hinge and results in higher antimicrobial activity and lower MICs. Work with these mutants supports the theory that helical structure is important for antimicrobial activity, and that mutations in the hinge region impact the antibacterial properties of the peptide.

To further investigate the importance of the hinge region, we tested how introduction of serine, a polar amino acid, or aspartic acid, a negatively charged amino acid, at position 24 would impact antimicrobial activity (CecBS and CecBD, respectively). In this case, we focused on characterizing the antimicrobial properties of CecBD and CecBS on *P. burhodogranarieaD*, *P. burhodogranarieaB*, and *P. sneebia* as these were the most sensitive to *D. melanogaster* cecropins overall. We found that for CecBD, antimicrobial activity was completely disrupted, with no inhibition of growth observed at our highest tested concentration (128 μM). This is unsurprising, as the introduction of a negatively charged amino acid in the hinge region likely disrupts favorable interactions with the negatively charged bacterial lipid membrane. While CecBS was unable to inhibit the growth of *P. sneebia* at any tested concentration, it displayed moderate activity against *P. burhodogranarieaD* (16 μM), an 8-fold increase in MIC compared to CecB (2 μM). Surprisingly, the activity of CecBS against *P. burhodogranarieaB* (4 μM) was comparable to that of CecB. In all cases, peptide activity against *P. burhodogranarieaD* seemed most sensitive to changes in the amino acid at position 24. Further, we find that when changing the chemical characteristics, especially from nonpolar to charged, the change in the MIC value no longer correlates with changes in helicity of the peptides in SDS. CecBD was measured to be slightly more helical than CecB, but has greatly reduced antimicrobial activity. This suggests that membrane disruption also depends on the general affinity of the peptide for the bacterial membrane. Altered affinity could potentially be due to the different composition of bacterial membranes of different species. Supporting this possibility, genome sequencing of the *Providencia* species reveals that genes unique to each species were enriched for membrane relevant gene ontology categories including pilus, cell adhesion, or cell projection.^44^

As all three cecropins can be expressed simultaneously postinfection in *D. melanogaster*,^33^ all native peptides were also tested in combination, both pairwise and with all three native peptides present from concentrations ranging between 2–64 μM total cecropin concentration. There was no additive or cooperative effect observed (data not shown). All peptides, native and mutated cecropins, were tested for cytotoxic effects when incubated with HeLa cells, and no substantial cytotoxicity was observed at any concentration tested, as high as 64 μM (Figure S4).

### Kinetics of Antimicrobial Activity

When determining the MIC of each peptide/bacteria pair, bacterial growth was measured after a 16-h incubation which does not provide insight into the speed of inhibition. We hypothesized that the alterations to the amino acid at position 24 might not only impact overall antimicrobial activity, but also the speed and mechanism by which the AMP inhibits bacterial growth. To assess the kinetics of activity, we investigated both the permeability and viability after treatment with an AMP over a much shorter time frame. Based on our previous results, we focused these kinetic experiments on the native CecA and CecB peptides along with the generated mutants that affect residue 24, CecAV, CecBD, CecBG, and CecBS to better understand the impact of the amino acid identity at this position. These peptides were assessed for the kinetics of their activity against *P. burhodogranarieaB*, *P. burhodogranarieaD*, and *P. sneebia* as the three *Providencia* species most susceptible to *D. melanogaster* cecropins. *P. rettgeri* and *P. alcalifaciens* were not investigated further as little/no growth inhibition was observed in MIC assays (Table 2).

**Figure 3:**
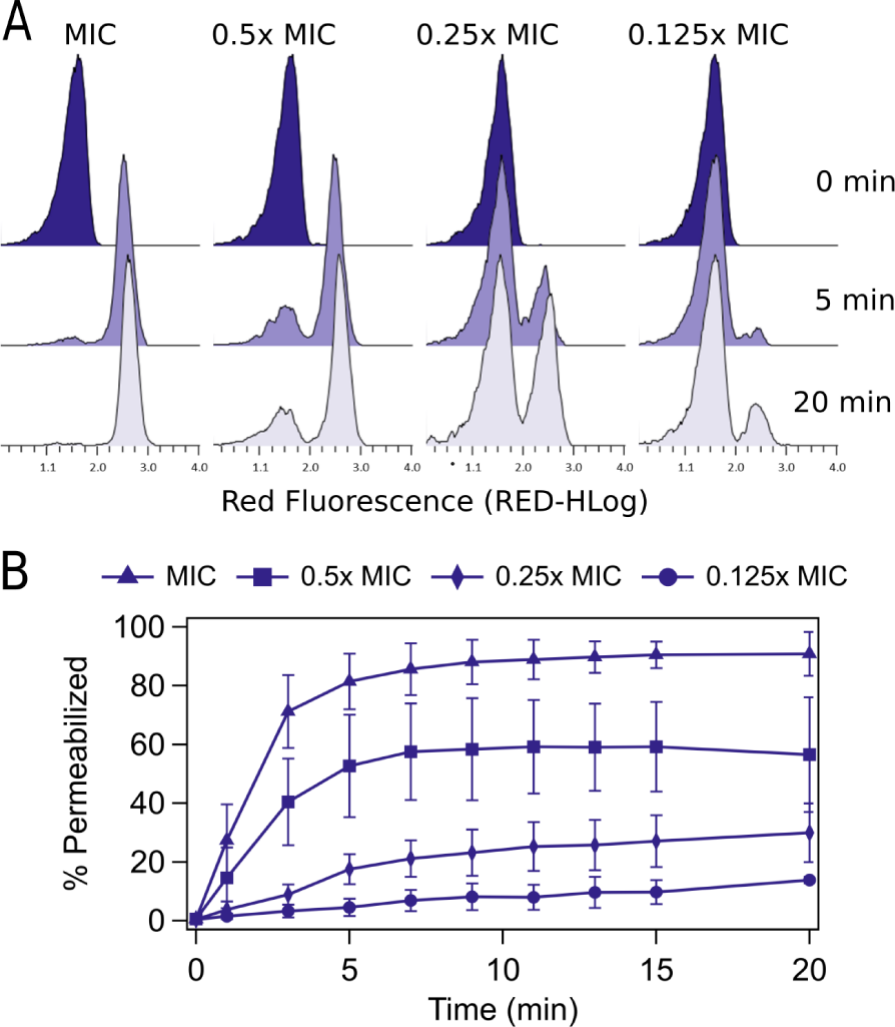
Kinetic analysis of cell permeabilization by AMPs. (A) Representative data to measure the population of *P. burhodogranarieaD* cells binding propidium iodide at 0, 5, and 20 min after treatment with CecB at MIC, 0.5× MIC, 0.25× MIC and 0.125× MIC. Overtime the number of cells that are permeable to propidium iodide increases even at concentrations below the MIC. (B) Percent of *P. burhodogranariea* D cells that are permeable to propidium iodide over time after treatment with CecB at MIC, 0.5× MIC, 0.25× MIC and 0.125× MIC. Each condition was repeated in triplicate and the graph depicts the average and standard deviation across these replicates. All other peptide-bacterial combinations show similar results and are available in Figure S6.

First, to assess permeability of the bacterial cells post-treatment, we used propidium iodide (PI), a dye that fluoresces when bound to DNA but is impermeable to intact cell membranes. Cells were analyzed by flow cytometry after various concentrations of an AMP were added to a bacterial culture; increasing cell fluorescence was indicative of a loss of membrane integrity, allowing PI to access the DNA within the bacterial cell (Figure 3). This enabled us to determine the proportion of cells that were permeabilized by AMP activity, but does not give a direct measure of cell viability. To directly assess the ability of bacterial cells to replicate and divide post-treatment, bacterial cultures were plated at various times after the addition of AMP. Colonies were counted after an overnight incubation to determine the number of replication competent (hence-forth called viable) cells, measured as colony forming units (CFUs). These experiments were performed using peptide concentrations at the previously determined MIC for each peptide-bacteria pair (Table 2).

**Figure 4:**
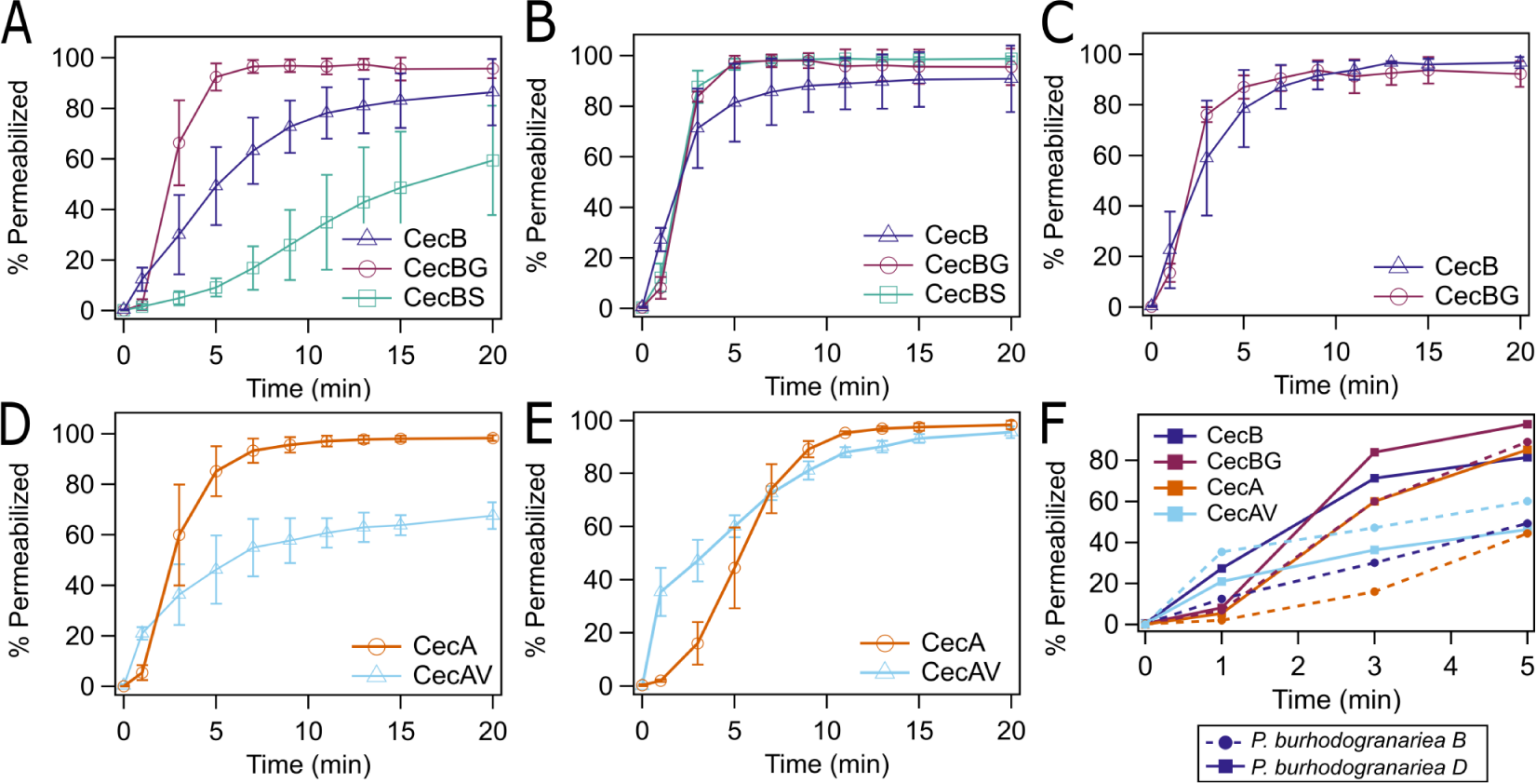
Amino acid changes in hinge impact kinetics of permeability most dramatically for *P. burhodogranarieaB*. (A) *P. burhodogranarieaB*, (B) *P. burhodogranarieaD* and (C) *P. sneebia* were treated with CecB, CecBG and CecBS at the determined MIC. (D) *P. burhodogranarieaB* and (E) *P. burhodogranarieaD* were treated with CecA and CecAV at the determined MIC. The percent permeability of intact cells was monitored for 20 min post-treatment using flow cytometry. *P. sneebia* is not depicted with CecBS as the MIC was greater than concentrations tested. (F) Enlargement of the first 5 min of the permeabilization curves, overlaying the data for the peptides containing Gly at position 24 (CecA, CecBG) and Val at position 24 (CecAV, CecB).

Overall, native CecB caused membrane permeability rapidly with all three bacterial species tested, with approximately 20% of cells permeable 1 min after addition of peptide at the MIC (Figure 3). Native CecA acted more slowly, only causing about 5% of cells to be permeable at 1 min after peptide addition (Figures 4D,E and S4). For both peptides, this fraction rapidly increased to greater than 80% by 7 min. When testing concentrations below the MIC, a rapid increase in cell permeabilization was also observed at 0.5× MIC, but a smaller overall portion of cells were observed to be permeable at 20 min. At significantly lower concentrations, i.e., 0.125× MIC, few cells became permeable even over 20 min (Figure S5). These experiments show that, at peptide concentrations below the MIC, a subset of cells is still susceptible to membrane permeabilization.

Although the MIC did not increase to a great extent when comparing CecB (2 μM) with CecBS (4 μM) and CecBG (8 μM) for *P. burhodogranarieaB*, we observed large differences in the kinetics of membrane permeabilization. This was especially apparent in the case of CecBS, where permeabilization occurred much more slowly compared to CecBG and CecB over 20 min (Figure 4A). After exposure to CecBG and CecBS at the MIC (32 and 16 μM, respectively), *P. burhodogranarieaD* had similar kinetics and overall permeability as when treated with native CecB (2 μM) (Figure 4B), even though the MIC had increased substantially. Similar kinetics and permeability were also observed with *P. sneebia* when comparing CecB and CecBG (CecBS was not tested due to the high MIC) (Figure 4C). In the presence of CecA (8 μM) and CecAV (4 μM), we again observed a large change in the rate of permeabilization over 20 min for *P. burhodogranarieaB* (Figure 4D). Consistent with the CecB mutants, the MIC for *P. burhodogranarieaD* was more greatly effected for the CecAV (2 μM) mutation compared to CecA (16 μM), but the speeds of permeabilization were nearly identical after approximately 7 min (Figure 4E). It is also notable that when peptides contained a Gly at position 24, a delay in permeability was observed at 1 min both for *P. burhodogranarieaB* and *P. burhodogranarieaD* when compared to peptides containing a Val at position 24 (Figure 4F). In most cases, the delay in permeabilization with the Gly peptides disappeared by 3 min. This data supports the hypothesis that the flexibility of the hinge impacts the kinetics of permeabilization. The large effect of a single amino acid in the hinge region on kinetics suggests that there might be something distinct about the *P. burhodogranarieaB* membrane-peptide interaction. Flow cytometry experiments were also performed with CecBD and *P. burhodogranarieaB* and *P. burhodogranarieaD* at a concentration of 128 μM, below the MIC, to determine whether this peptide had any effect on permeability. After 20 min, less than 30% of cells were permeable for both bacterial strains (Figure S5G,H), similar to experiments performed with other peptides at values below the MIC. With the incorporation of the negatively charged Asp in the hinge region, CecBD is still capable of causing membrane disruption, but much higher concentrations of peptide are required.

**Figure 5:**
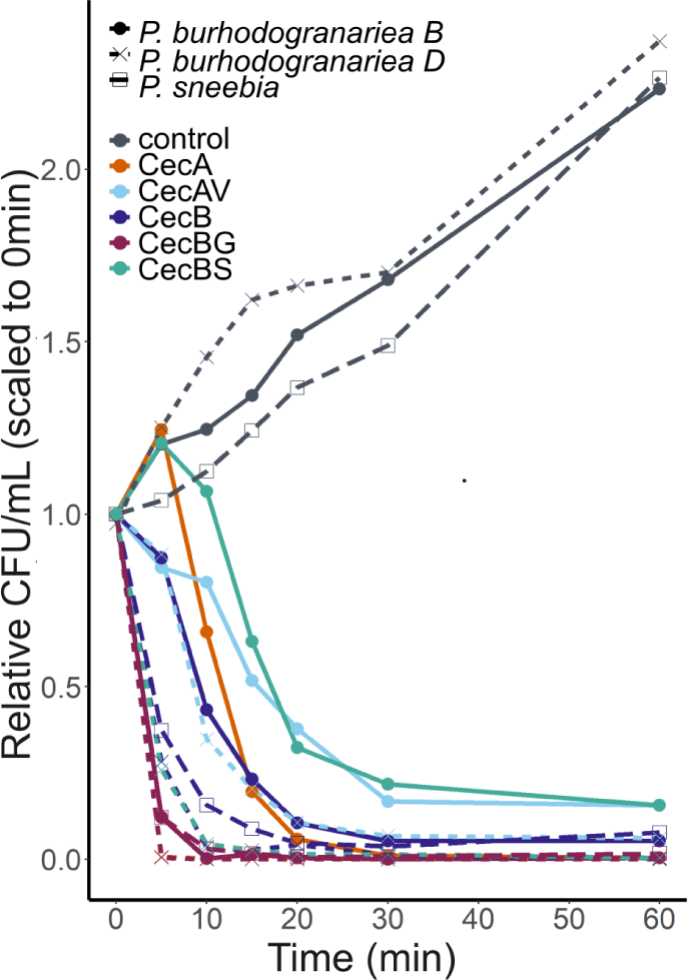
Impact of CecA and CecB variants on kinetics of cell viability. Relative number of bacterial cells capable of forming colonies over time after exposure to the MIC for CecA, CecAV, CecB, CecBG and CecBS. All three bacteria exposed to the MIC for a given peptide had very few cells capable of producing colonies by 20 min post treatment. However, *P. burhodogranarieaB* bacterial cells retained the ability for form colonies for longer across all peptides tested except CecBG where kinetics resembled the other two bacterial species. To account for variation in starting number of bacterial cells between assays, all values were scaled to the number at time zero within that vial. Examples of nonscaled assays are in Figure S6.

Similar trends to the cell permeabilization experiments were observed when measuring kinetics of viability using a CFU assay, in which *P. burhodogranarieaB* cells lost viability more slowly with most peptides relative to the other two bacterial species (Figures 5 and S6), primarily in the first 5 min of incubation with peptides. In comparison, the number of CFUs for both *P. burhodogranarieaD* and *P. sneebia* rapidly decreased in the first 5 min. Loss of viability was slowest for *P. burhodogranarieaB* treated with both CecA and CecBS, although these peptides were able to swiftly kill both *P. burhodogranarieaD* and *P. sneebia*. In all cases, the CFUs decreased to nearly undetectable levels within 30 min.

**Figure 6:**
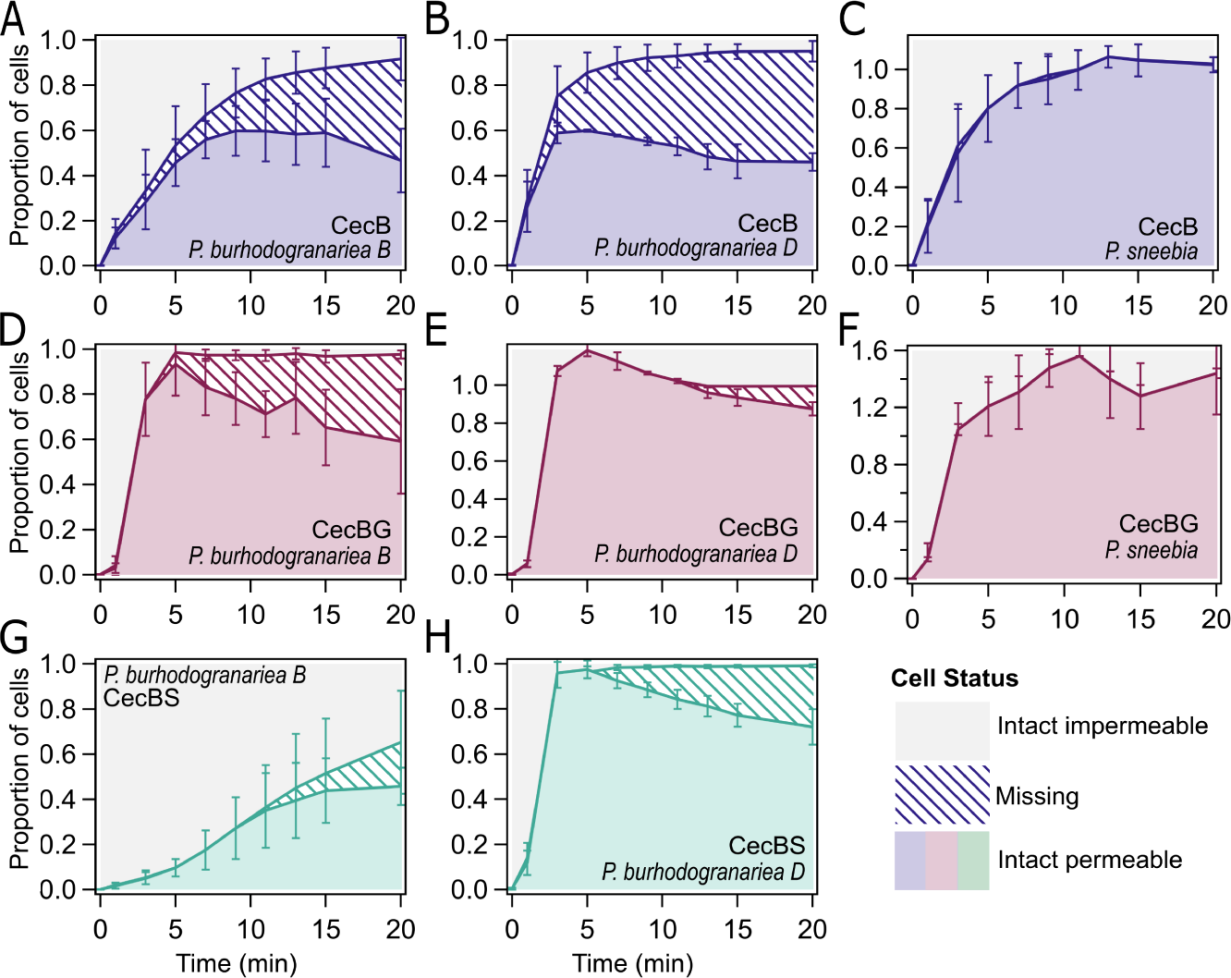
Bacterial species and amino acid at position 24 impact disappearance and viability of bacterial cells (A–G) Visualization of cell populations that are impermeable (gray), missing (hatched), or permeable (solid color) after treatment at MIC (A) *P. burhodogranarieaB* with 2 μM CecB, (B) *P. burhodogranarieaD* with 2 μM CecB, and (C) *P. sneebia* with 32 μM CecB. (D) *P. burhodogranarieaB* with 8 μM CecBG, (E) *P. burhodogranarieaD* with 32 μM CecBG, and (F) *P. sneebia* with 128 μM CecBG. (G) *P. burhodogranarieaB* with 4 μM CecBS, (H) *P. burhodogranarieaD* with 16 μM CecBS. Data for CecA and AV is in Figure S6.

**Figure 7:**
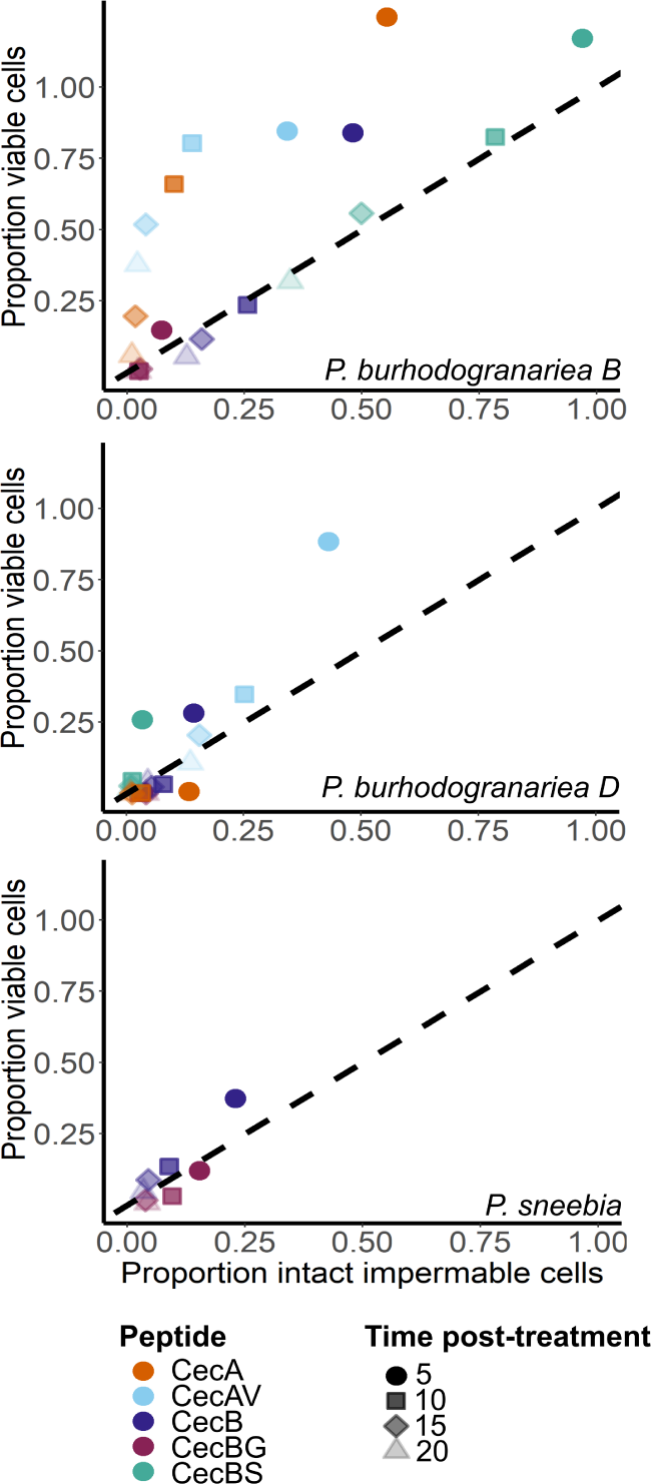
Subset of permeabilized cells remain temporarily viable. Relationship between proportion of bacterial cells capable of forming colonies and impermeable cells over time after exposure to the MIC for CecA and CecB variants. Dashed line indicates where data would fall if the proportion capable of forming colonies equaled that of intact impermeable cells. To account for variation in starting number of bacterial cells between assays, all values were scaled to the number at time zero within treatment.

To further corroborate this trend, total cell counts as measured by flow cytometry were analyzed to determine the relative populations of impermeable, permeable, and missing cells. Missing cells were attributed to loss of cell morphology due to action of the AMPs, and calculated by the decrease in cell concentration compared to the concentration of cells immediately before the addition of peptide. Approximately 60% of *P. burhodogranarieaB* cells were missing after 20 min of incubation with CecB (Figure 6A), while 50% of *P. burhodogranarieaD* cells were missing (Figure 6B). Conversely, almost no *P. sneebia* cells were missing even after 20 min of incubation (Figure 6C); in this case, the cells were still not viable as determined by the lack of colonies of agar plates, and were highly permeable, but were intact enough to be detected with the same size parameters by flow cytometry as the untreated cells. Similar results were observed with CecA (Figure S7). This suggests that the bacterial species vary in their robustness after treatment. *P. sneebia* cells appear to have a very robust cell envelope architecture, as cells remain intact even though they have been permeabilized and no longer are capable of producing colonies. This feature could be responsible for the low immunogenicity of *P. sneebia* as immune stimulation may be partially due to microbial associated compounds that are released when cells are lysed. *P. burhodograrieaB*, in contrast, appears to be more resilient to permeabilization as the proportion of cells able to form colonies is higher than the proportion of impermeabilized cells (Figure 7), suggesting that, despite permeabilization, cells are able to replicate successful when plated. In contrast, both *P. burhodogranarieaD* and *P. sneebia* had similar proportions of cells capable of forming colonies and impermeable cells, suggesting that once permeabilized they lose their ability to replicate and form colonies.

Based on MIC data, *P. burhodogranarieaD* was extremely sensitive to changes in the amino acids at position 24 with any change away from the native CecB Val residue greatly curtailing activity. While sensitivity to this position might have suggested that CecBG and CecBS would act more slowly, we found that as long as bacterial cells were treated at the MIC, kinetics matched those of CecB, just requiring higher peptide concentrations. In contrast, while *P. burhodogranarieaB* seemed less sensitive to changes in the amino acid at position 24 based on MIC, kinetics of permeability and viability were more dramatically impacted by changing the residue at this position. The largest change was observed with CecBS, which switches the residue from a nonpolar to a polar amino acid.

## Conclusions

When considering all of the above results, it is clear that changing a single amino acid can dramatically alter the antibacterial activity of cecropins while having only minimal impacts on overall structure of the peptide. When amino acid mutations are relatively conservative (i.e., Gly to Val), we observed small increases in the helicity of the peptide, and the amount of helicity measured in the presence of both SDS and PC compared to TFE, that correlates with more effective antibacterial activity. This correlation was not observed when the chemical characteristic of the amino acid side chain was altered (i.e., Val to Asp or Ser). We hypothesize that the additional flexibility of the hinge region with Gly at position 24 can impede antibacterial activity. We have discovered two different ways by which activity can change, depending on the specific interactions between the AMP and the bacteria, potentially due to differences in bacterial membrane composition between species. For both *P. sneebia* and *P. burhodogranarieaD*, changing the amino acid identity at position 24 in the AMP hinge region changed the overall MIC, much more dramatically in the case of *P. burhodogranarieaD*, but the kinetics of action, when measured at the MIC, did not display notable differences. The MIC of the peptide mutants when tested with *P. burhodogranarieaB* showed much smaller changes, but the rate of permeabilization and loss of cell viability measured at the MIC for each peptide showed much larger variation. This indicates that, although *P. burhodogranarieaB* is still susceptible to the cecropin mutants, the speed of action changes. We can conclude that specific interactions between the bacterial membrane and the peptides dictate antimicrobial activity, although work is still needed to determine what components of the membrane control these interactions.

## Methods

### Bacterial Species

This work used five *Providencia* species that were isolated from field caught *D. melanogaster* (obtained from Brian Lazzaro).^31^ The bacteria from this genus were chosen for this study due to the fact that *Providencia* species have a range of virulence with *D. melanogaster*.^32^ Bacterial stocks were maintained as described previously.^45,46^ Briefly, bacterial stocks were stored long-term at −80 °C in Luria–Bertani (LB) broth containing 15% glycerol. Bacteria were first streaked on an LB agar plate from the stock, grown overnight at 25 °C, and subsequently stored at 4 °C for up to 1 week.

### Peptide Synthesis and Purification

Peptides were synthesized using a Gyros Protein Technologies PS3 peptide synthesizer (Sweden) with standard Fmoc (9-fluorenylmethoxycarbonyl) chemistry used for synthesis. Fmoc-Rink Amide resin (Aapptec, USA) was used for all syntheses, resulting in an N-terminal amide group, and standard protecting group chemistry was used for each amino acid (Aapptec, USA). Briefly, the Fmoc protecting group on the peptide chain was removed using a solution of 20% (v/v) piperidine in dimethylformamide (DMF). A 4-fold excess (in relation to the molar loading capacity of the resin) of each amino acid and an equimolar amount of 2-(1H-benzotriazol-1-yl)-1,1,3,3-tetramethyluronium (HBTU) were dissolved in 0.4 M *N*-methylmorpholine in DMF, added to the peptide resin, and the coupling reaction proceeded for 20 min with agitation by bubbling with nitrogen gas. The resin was washed with DMF between each step, and the synthesis proceeded until all amino acids were incorporated. The N-terminal Fmoc protecting group was removed using a solution of 20% piperidine in DMF.

The peptide resin was dried *in vacuo* and the peptide was cleaved from the resin using a solution of 5% (v/v) thioanisole, 5% (w/v) phenol, 5% (v/v) water, and 2.5% (v/v) ethane dithiol in trifluoroacetic acid (TFA) for 1.5–2 h at room temperature with stirring. The peptide solution was filtered using a sintered glass filter to remove the resin and was then added to a 10x volume of diethyl ether. This solution was chilled at −80 °C for at least 1 h before being filtered. The peptide was collected as a solid, washed with additional cold ether, redissolved in a solution containing 20% acetonitrile in water, filtered using a 0.2 μm syringe filter to remove any precipitate, and purified via preparative-scale High Pressure Liquid Chromatography (HPLC).

A Shimadzu SPD-10A or Agilent 1260 Infinity II HPLC was used for reverse-phase preparative-scale HPLC with a Phenomenex Luna 10 μm PREP C18(2) 100 Å column. Water containing 0.1% TFA was used as solvent A and acetonitrile was used as solvent B with a flow rate of 9.99 mL/min. Peptide absorbance was monitored at 230 nm, and fractions were collected manually. The purity of fractions containing the desired peptide was verified by analytical-scale HPLC using an Agilent Technologies 1260 Infinity HPLC equipped with an Agilent Eclipse Plus C18 column; all peptides were verified to be >90% pure before additional experiments were run (Figure S2). The analytical HPLC protocol used the same solvents as buffer A and B as preparative-scale HPLC, but with a flow rate of 1.00 mL/min.

Electrospray ionization mass spectrometry (ESI-MS) was conducted using an Advion Expression-L compact mass spectrometer (Advion Interchim Scientific, USA) in positive ion mode. Peptide samples were dissolved to a concentration of approximately 0.05 mg/mL in a solution of 20–50% acetonitrile in water before analysis. Approximately 5 μL aliquots were analyzed by flow injection analysis into 100% HPLC-grade acetonitrile at 0.2 mL/min to confirm the mass of each synthesized peptide (Figure S2).

### Analysis of Peptide Secondary Structure

#### Circular Dichroism (CD) Spectroscopy

CD spectra were measured using a Jasco J-1500 circular dichroism spectrophotometer with 1 cm path length quartz cuvettes. The absorbance of each sample was measured from 260 to 190 nm with a 0.1 nm data pitch, a 1.00 nm bandwidth, and a 1.0 s integration time. Each spectrum was collected 3 times and the signals were averaged with the temperature maintained at 25 °C. Peptides were prepared at a final concentration of 2.5 μM in 10 mM sodium phosphate buffer, pH 7.1. For samples containing sodium dodecyl sulfate (SDS), the final concentration of SDS in the peptide sample was 30 mM. For samples containing trifluoroethanol (TFE), the final concentration of TFE in the peptide solution was 50%. For samples containing Soy L-α-phosphatidylcholine (PC), the final concentration of PE in the peptide sample was 0.5 μM, as concentration was limited by light scattering. All samples were prepared with a final volume of 1.5 mL in a 1 cm path length quartz cuvette.

#### Peptide Modeling

Peptide structures were modeled using the I-TASSER structure prediction server.^34−36^

#### Analysis of Cytotoxicity

HeLa cells were cultured in a 96-well plate and grown for 48 h until they reached ∼90% confluence. Peptides were then added to separate wells in triplicate at concentrations of 64 μM, 32 μM, 16 μM, or 8 μM and incubated at 37 °C for 24 h. Twelve wells were used as a positive control, where no peptide was added. A solution of 3-[4,5-dimethylthiazol-2-yl]-2,5 diphenyl tetrazolium bromide (MTT) was then added to each well to a final concentration of 0.45 mg/mL and incubated for 3 h at 37 °C. A 1:1 volumetric ratio of solubilizing solution (40% v/v dimethylformamide, 2% v/v glacial acetic acid, 16% w/v sodiumdodecylsulfate in water) was added to each well. The absorption at 570 nm was measured using a BioTek Synergy H1Microplate Reader.

### In Vitro Antimicrobial Activity of *Drosophila* Cecropins

#### Bacterial Growth Conditions

For all antimicrobial testing, bacteria were cultured in order to obtain cells in their exponential growth phase. Liquid bacterial cultures were inoculated from a single bacterial colony on a streaked LB agar plate. Cultures were grown overnight in Mueller Hinton (MH) broth in an orbital shaker at 150 rpm for 16 h at 25 °C. The optical density (Absorbance_600 nm_) of overnight cultures were determined using a spectrophotometer (SmartSpec Plus, Bio-Rad, USA or Wireless Spectrometer (Vis), PASCO, USA). The overnight culture was then diluted to an optical density of 0.002 in M–H broth and incubated at 25 °C with shaking (150 rpm). Bacterial growth curves were determined experimentally for each bacterial species and at each temperature (Figure S4). Subcultures were grown for 5 h to ensure that each subculture was in its logarithmic growth phase and at a culture density above 0.1 Abs_600 nm._ After growth of the subculture for the designated time, the optical density was again measured and the bacterial culture was diluted to 0.002 Abs_600 nm_ (∼2 × 10^6^ CFU/mL) in M–H broth for use in subsequent assays.

#### Minimum Inhibitory Concentration (MIC) Assay

The MIC for each peptide was determined using microdilution antimicrobial testing in 96-well plates. Peptide stocks were prepared at 1.28 mM in water and diluted to the desired concentration using M–H broth. A serial dilution was performed, with final peptide concentrations ranging from 64–1 μM or 128–2 μM on each plate. The plate was incubated on an orbital shaker (150 rpm) at 25 °C for 16 h. Growth inhibition was determined by measuring the optical density at 600 nm using a BioTek Synergy H1Microplate Reader. The MIC was determined to be the lowest concentration of peptide where there was no detectable difference in optical density when compared to wells lacking bacteria.

#### Permeability Assay

Bacterial cultures were treated with peptides at the experimentally determined MIC, 0.5× MIC, 0.25× MIC, and 0.125× MIC. Samples were prepared containing 5 μg/mL propidium iodide (PI) in MH broth. The cells were assessed by flow cytometry using a Millipore Guava easyCyte 8 at 0, 1, 3, 5, 7, 9, 11, 13, 15, and 20 min post-treatment and were incubated at 25 °C between measurements. Control samples containing PI without peptide were prepared for each trial; no changes were observed for these controls over the course of the experiment. Data processing was performed using OMIQ. Briefly, gates were set based on the initial scattering profiles of cells. A threshold was determined for red fluorescence; cells above that threshold were determined to be permeable. Gates and thresholds were applied consistently to each sample.

The number of total cells dropped over time for many samples due to peptide treatment, and the number of total cells at each time-point were scaled to the number of cells at 0 min. The number of destroyed or “missing” cells was determined by calculating the number of permeable and impermeable cells present at a given time, and subtracting those values from the total number of cells at 0 min.

#### Viability Assay

Bacterial cultures were treated with peptides at the experimentally determined MIC and small aliquots were removed at 0, 5, 10, 15, 20, 30- and 60 min post-treatment and diluted 100-fold in ice cold M–H broth. All zero-minute time-points were removed within 20 s of peptide being added. Diluted aliquots were then plated on M–H agar plates using a WASP II autoplate spiral plater (Microbiology International), which deposits 50 μL on each plate in a continuously decreasing concentric spiral. Plates were grown for 36 h at 25 °C and then counted using the EZ-Count Automated Colony Counter (Microbiology International), which calculates the number of Colony Forming Units (CFU) per mL based on the number of colonies and their position along the spiral, allowing for resolution of 3 orders of magnitude on a single plate. All aliquots were plated three times, and CFU counts averaged between the replicates and examples of a single assay for each species and peptide combination is in Figure S2. In order to compare across species and independent assays, all counts were then scaled to the average at zero minutes post-treatment.

#### Data Visualization

Data were visualized using either Igor Pro (9.00) or the ggplot2 package (v3.4.1; Wickham, 2016) and panels assembled using InkScape.

## Supporting Information Available

Analysis of peptide purity supporting data including bacterial growth curves, mammalian cell cytotoxicity, additional kinetic data for individual bacteria/peptide combinations (PDF)

## Abbreviations

*D. melanogaster*: *Drosophila melanogaster*
AMP: antimicrobial peptide
*P. rettgeri*: *Providencia rettgeri*
*P. burhodogranariea**B*: *Providencia burhodogranariea**B*
*P. burhodogranariea**D*: *Providencia burhodogranariea**D*
*P. sneebia*: *Providencia sneebia*
*P. alcalifaciens*: *Providencia alcalifaciens*
TFE: trifluoroethanol
PI: propidium iodide
LB: Luria Burtani
HBTU: 2-(1*H*-benzotriazol-1-yl)-1,1,3,3-tetramethyluronium
M–H: Mueller–Hinton
CFU: colony forming unit
