# Supplementary material for: Single Amino Acid Changes Impact the Ability of Drosophila melanogaster Cecropins to Inhibit Growth of Providencia Pathogens
Source: ACS Omega. 2025 Feb 5;10(6):5403–14. doi: 10.1021/acsomega.4c07262 (PMC11840601; doi:10.1021/acsomega.4c07262)
Supplement: Supplementary file 1 — ao4c07262_si_001.pdf [file ao4c07262_si_001.pdf]

Supporting information for

Single amino acid changes impact the ability of *Drosophila melanogaster* cecropins to inhibit growth of *Providencia* pathogens

Marla J. Forfar,[1] Christopher R. Feudale,[2] Lauren E. Shaffer,[3] Grace M. Ginder,[3] Marion E. Duval,[2] Michelle Vovsha,[3] Quinn B. Smith,[4] Moria C. Chambers,[2,3*] Sarah J. Smith[1,2*]

[1]Department of Chemistry, Bucknell University
[2]Program in Cell Biology and Biochemistry, Bucknell University
[3]Department of Biology, Bucknell University
[4]Program in Neuroscience, Bucknell University

***Email: sarah.smith@bucknell.edu, moria.chambers@bucknell.edu**

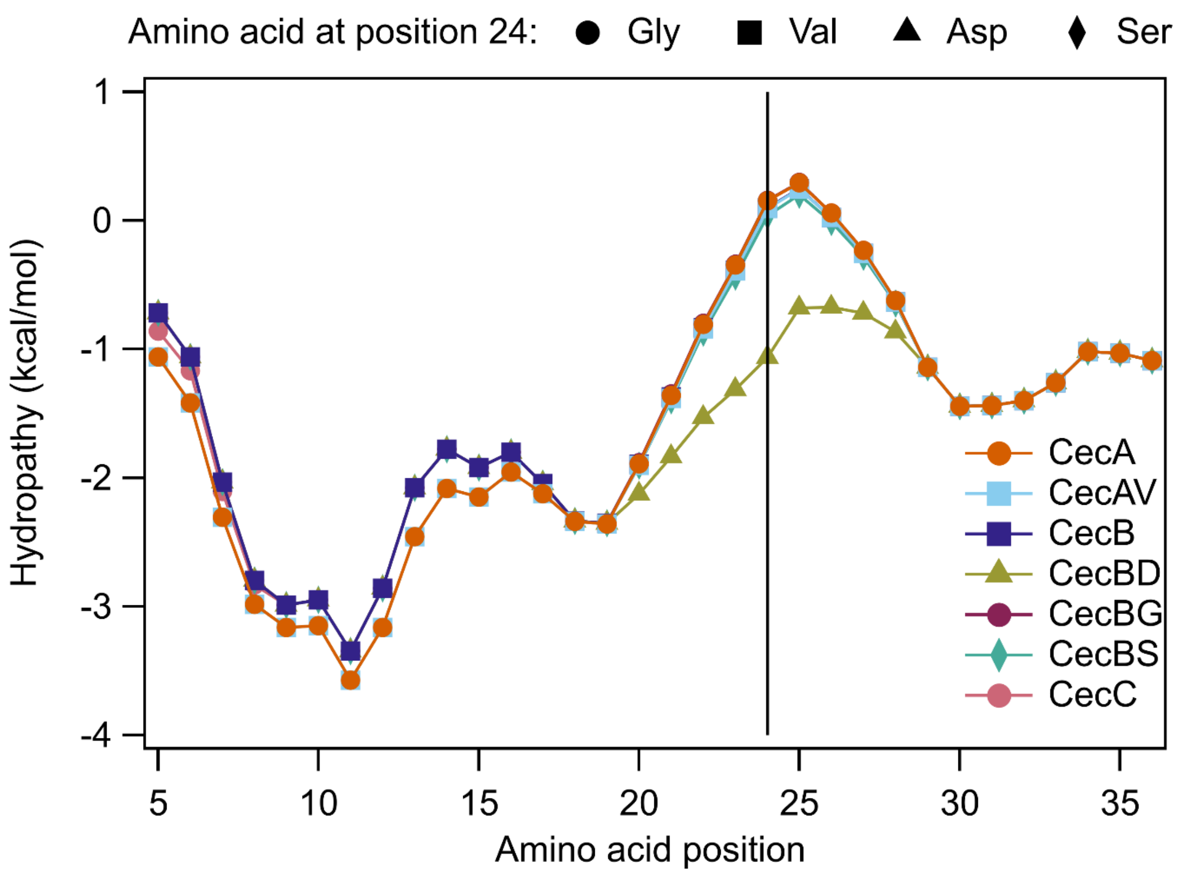

Figure S1. All Cecropins except CecBD have nearly identical hydropathy profiles. The hydropathy of each peptide was analyzed using MPEx in Scan mode with a window size of 5. The profiles for CecA and CecAV overlay, as do the profiles for CecB, CecBG, CecBS, and CecC. The line at position 24 indicates the position of the mutation in the expected hinge region. CecBD is different, due to the inclusion of the negatively charged Asp residue at position 24.

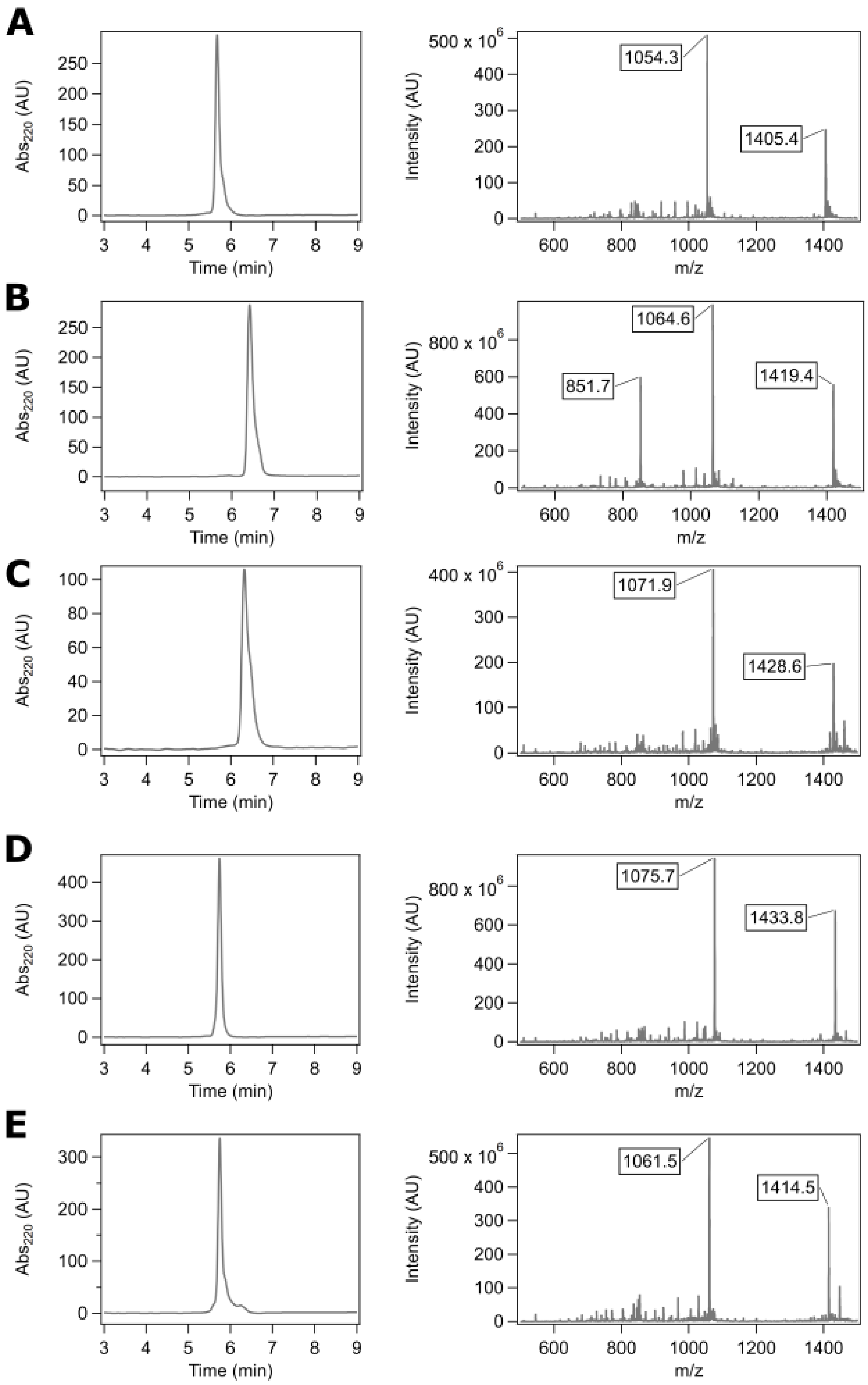
A
B
C
D
E
Abs220 (AU)
Time (min)
Intensity (AU)
m/z
500 x 10^6
800 x 10^6
400 x 10^6
1054.3
1405.4
1064.6
851.7
1419.4
1071.9
1428.6
1075.7
1433.8
1061.5
1414.5

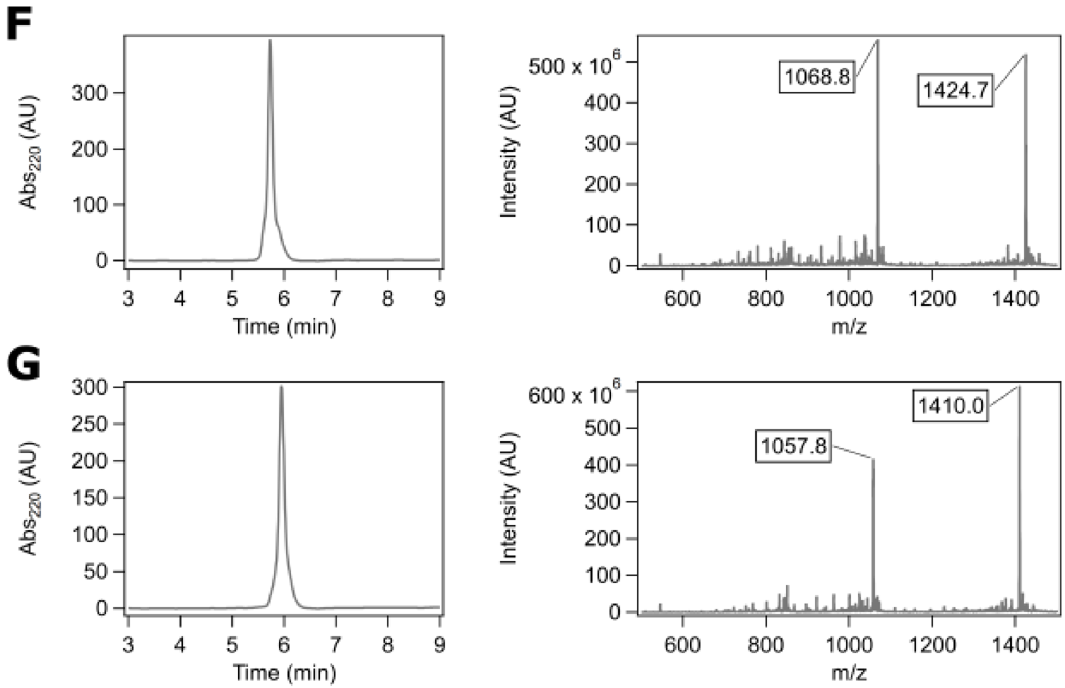

Figure S2. Cecropin peptide analysis via analytical HPLC and ESI-MS. HPLC conditions: solvent A 0.1% TFA in water, solvent B acetonitrile. HPLC Gradient: 5% to 70% solvent B over 10 min, 1.00 mL/min flow rate. (a) CecA: Expected mass: 4212.8 g/mol. Observed: 1405.4 $[M+3H]^{3+}$, 1054.3 $[M+4H]^{4+}$. (b) CecAV: Expected mass: 4254.9 g/mol. Observed 1419.4 $[M+3H]^{3+}$, 1064.6 $[M+4H]^{4+}$, 851.7 $[M+5H]^{5+}$. (c) CecB: Expected mass: 4282.9 g/mol. Observed: 1428.6 $[M+3H]^{3+}$, 1071.9 $[M+4H]^{4+}$. (d) CecBD: Expected mass: 4298.9 g/mol. Observed: 1433.8 $[M+3H]^{3+}$, 1075.7 $[M+4H]^{4+}$. (e) CecBG: Expected mass: 4240.8 g/mol. Observed: 1414.5 $[M+3H]^{3+}$, 1061.5 $[M+4H]^{4+}$. (f) CecBS: Expected mass: 4270.9 g/mol. Observed: 1424.7 $[M+3H]^{3+}$, 1068.8 $[M+4H]^{4+}$. (g) CecC: Expected mass: 4226.9 g/mol. Observed: 1410.0 $[M+3H]^{3+}$, 1057.8 $[M+4H]^{4+}$.

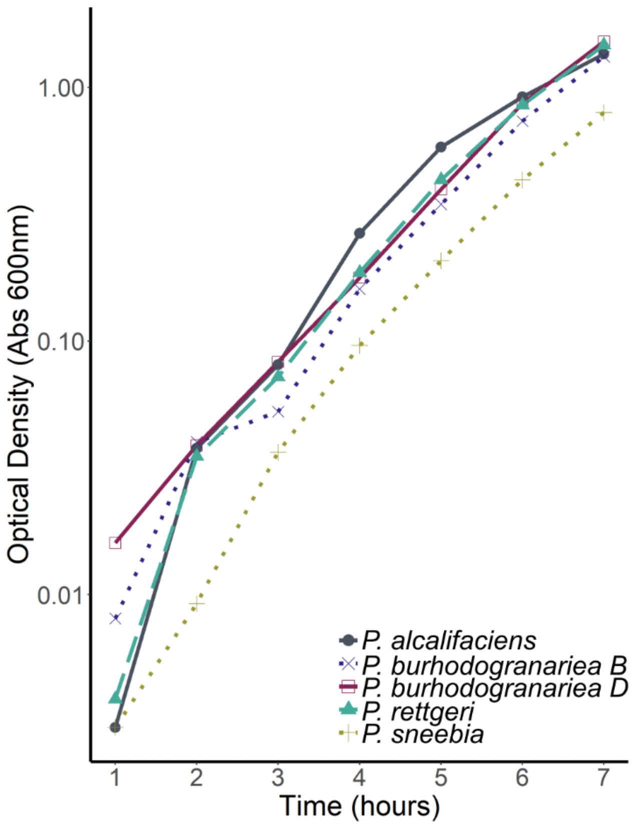

Figure S3. Growth kinetics of bacterial subcultures at 25°C. Overnight cultures grown for 16 hours at 25 °Cwere used to inoculate 50 mL flasks to generate a starting optical density of 0.002 (Abs 600nm). Absorbance was measured hourly using a spectrophotometer to determine optimal timing for both measurable densities and exponential growth phases. Each strain was assessed in duplicate at each temperature and depicted are the average values from the two replicates. Bacteria were typically used for assays around 5 hours post-subculture when bacteria were in exponential growth phase and had optical densities above 0.1.

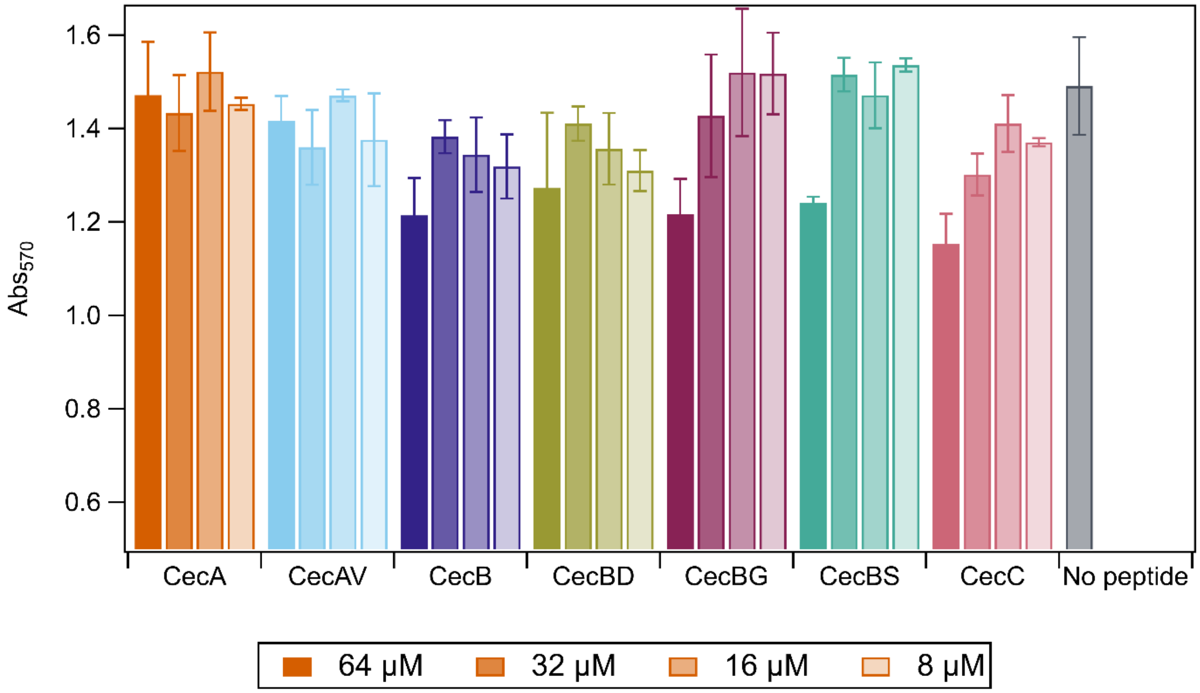

Figure S4. Analysis of peptide cytotoxicity after 24 hr incubation with HeLa cells. HeLa cells were grown to 90% confluence, incubated with Cecropin peptide at the noted concentration for 24 hr, and an MTT assay was then performed to measure the cytotoxic effects of the peptides. No substantial cytotoxicity was observed with any of the peptides.

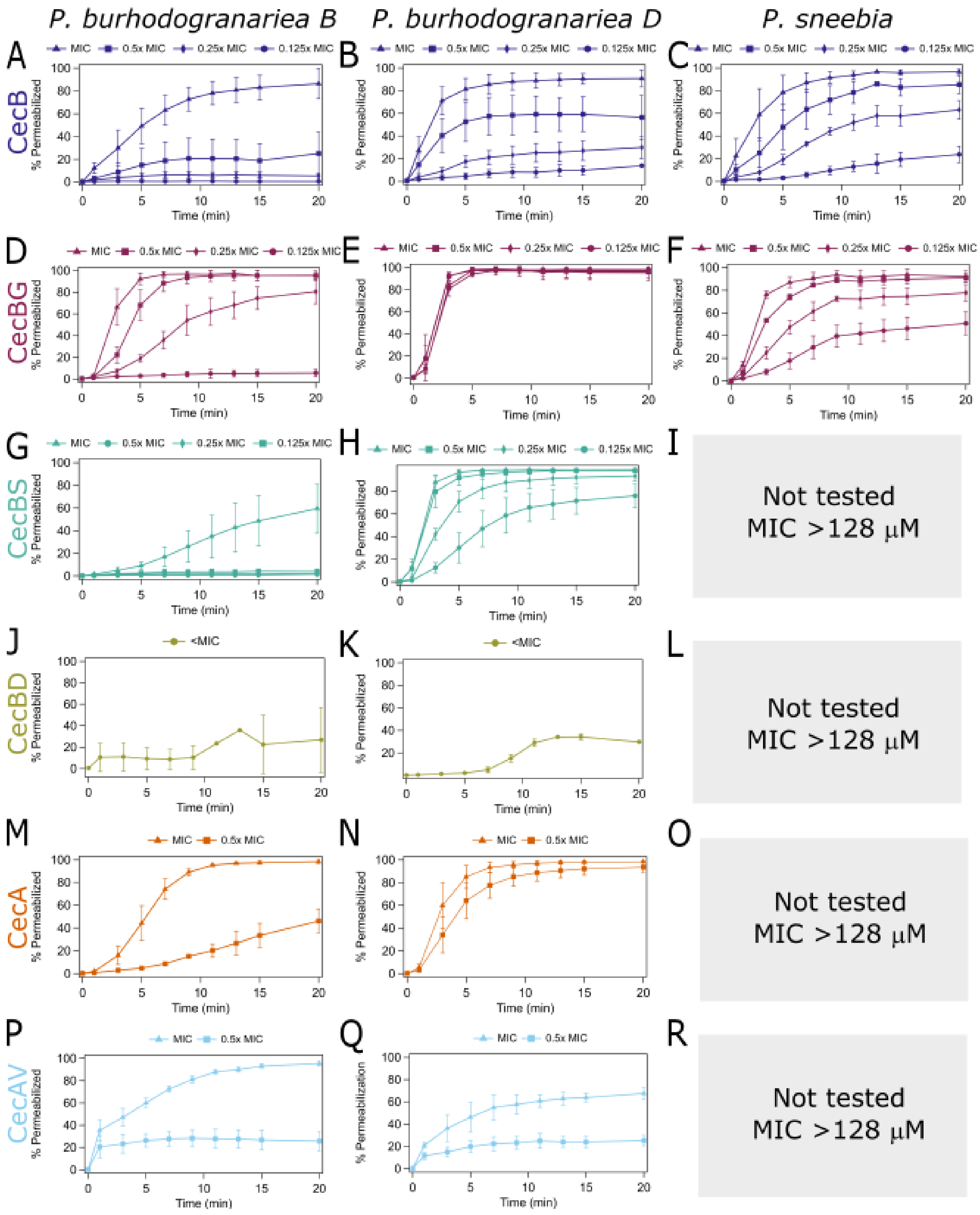

P. burhodogranariea B
P. burhodogranariea D
P. sneebia
A
B
C
CecB
MIC
0.5x MIC
0.25x MIC
0.125x MIC
% Permeabilized
Time (min)
D
E
F
CecBG
G
H
I
CecBS
Not tested
MIC >128 μM
J
K
L
CecBD
<MIC
Not tested
MIC >128 μM
M
N
O
CecA
Not tested
MIC >128 μM
P
Q
R
CecAV
% Permeabilization
Not tested
MIC >128 μM

Figure S5. Impact of CecB variants on permeability at and below MIC. (A-R) Percent of bacterial cells that are permeable to propidium iodide over time after treatment with the given peptide at MIC, 0.5x MIC, 0.25x MIC and 0.125x MIC. Each condition was repeated in triplicate and the graph depicts the average and standard deviation across these replicates. (A) *P. burhodogranariea B*, MIC is 2 μM CecB, (B) *P. burhodogranariea D*, MIC is 2 μM CecB, and (C) *P. sneebia*, MIC is 32 μM CecB. (D) *P. burhodogranariea B*, MIC is 8 μM CecBG, (E) *P. burhodogranariea D*, MIC is 32 μM CecBG, and (F) *P. sneebia*, MIC is 128 μM CecBG. (G) *P. burhodogranariea B*, MIC is 4 μM CecBS, (H) *P. burhodogranariea D*, MIC is 16 μM, CecBS. (I) *P. sneebia* was not tested with CecBS, (J) *P. burhodogranariea B* and (K) *P. burhodogranariea D* with 128 μM, which is below the MIC. (L) *P. sneebia* was not tested with CecBD. (M) *P. burhodogranariea B*, MIC is 8 μM CecA. (N) *P. burhodogranariea D*, MIC is 16 μM CecA. (O) *P. sneebia* was not tested with CecA. (P) *P. burhodogranariea B*, MIC is 4 μM CecAV. (Q) *P. burhodogranariea D*, MIC is 2 μM CecAV. (R) *P. sneebia* was not tested with CecAV.

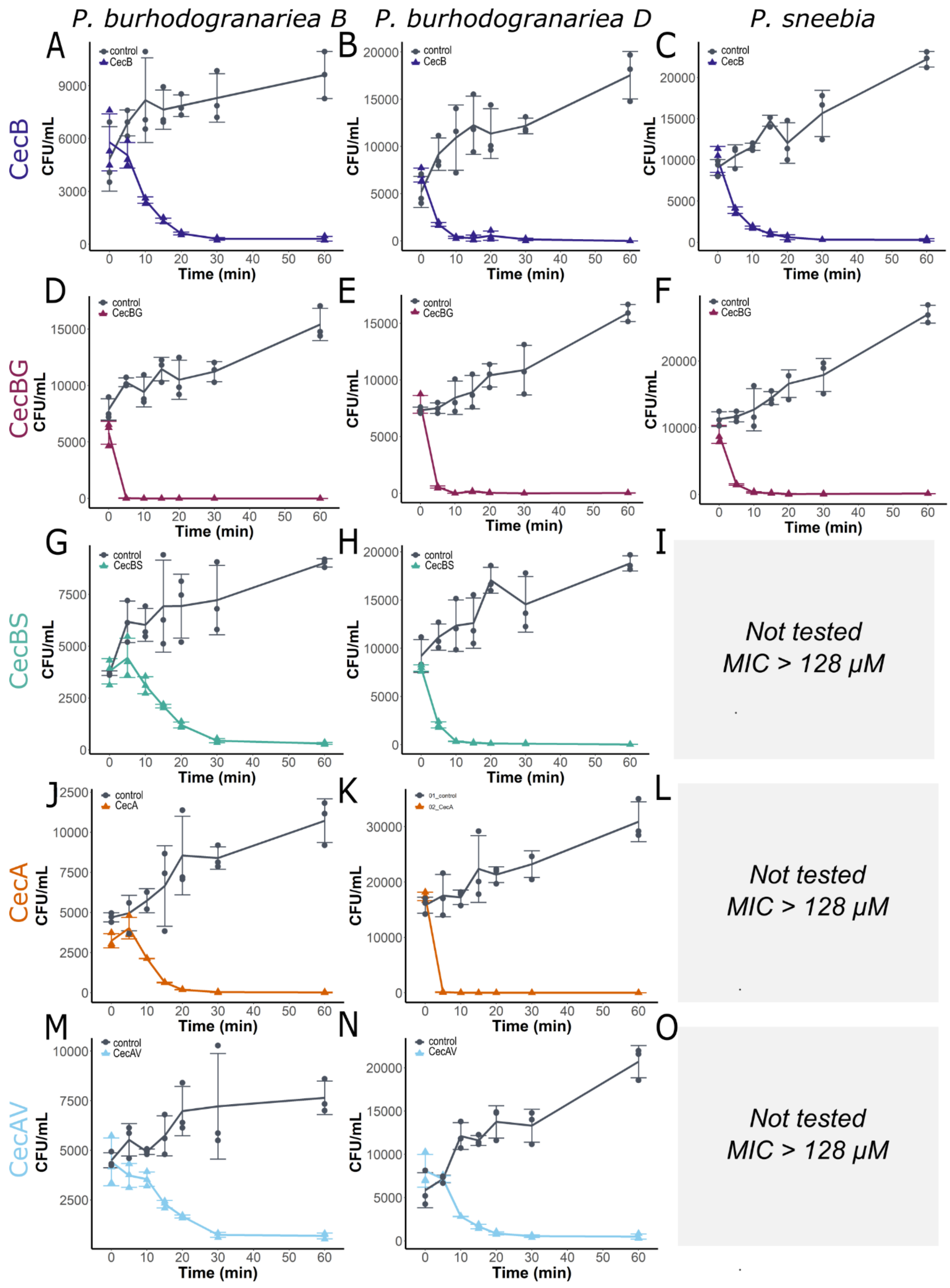

P. burhodogranariea B
P. burhodogranariea D
P. sneebia
A
B
C
D
E
F
G
H
I
J
K
L
M
N
O
CecB
CecBG
CecBS
CecA
CecAV
control
CecB
CecBG
CecBS
CecA
CecAV
01_control
02_CecA
CFU/mL
Time (min)
Not tested
MIC > 128 μM
Not tested
MIC > 128 μM
Not tested
MIC > 128 μM

Figure S6. Number of bacterial cells capable of forming colonies over time after exposure to the MIC for CecB variants. (A, D, G, J & M) *P burhodogranariea B*, (B, E, H, K & N) *P. burhodogranariea D*, and (C & F) *P. sneebia* were treated with either (A-C) CecB, (D-F) CecBG , (G-H) CecBS, (J-K) CecA, or (M-N) CecAV at the MIC for each bacterial-peptide combination: (A) *P burhodogranariea B* with 2 µM CecB, (B) *P burhodogranariea D* with 2 µM CecB, (C) *P sneebia* with 32 µM CecB, (D) *P burhodogranariea B* with 8 µM CecBG, (E) *P burhodogranariea D* with 32 µM CecBG, (F) *P sneebia* with 128 µM CecB, (G) *P burhodogranariea B* with 4 µM CecBS, (H) *P burhodogranariea D* with 16 µM CecBS, (I) *P. sneebia* was not tested with CecBS as the MIC was greater than 128 µM, (J) *P burhodogranariea B* with 8 µM CecA, (K) *P burhodogranariea D* with 16 µM CecA, (L) *P. sneebia* was not tested with CecA as the MIC was greater than 64 µM, (M) *P burhodogranariea B* with 2 µM CecAV, (N) *P burhodogranariea D* with 2 µM CecAV, (O) *P. sneebia* was not tested with CecAV. Viability of bacterial cells was assessed using colony forming unit assays, and at each time point three replicates were done for every condition. Each dot represents the CFU/mL as determined by a single plate, lines pass through the average CFU/mL and error bars reflects the standard deviation. All three bacteria exposed to the MIC for a given peptide had very few cells capable of producing colonies by 20 minutes post treatment, however, *P. burhodogranariea B* bacterial cells retained ability to form colonies for longer with similar number of cells capable of forming colonies as controls at 5 minutes post-exposure.

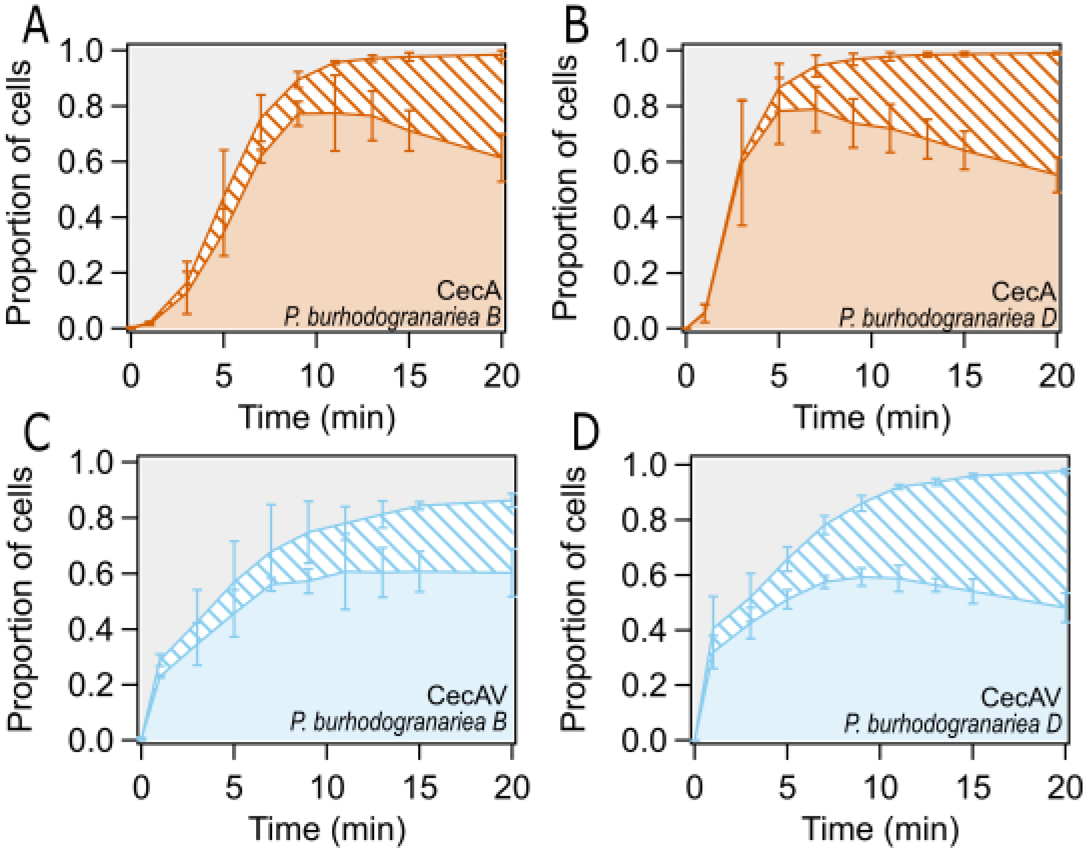

Figure S7. Bacterial species and amino acid at position 24 impact disappearance and viability of bacterial cells (A-D) Visualization of cell populations that are impermeable (grey), missing (hatched), or permeable (solid color) after treatment at MIC (A) *P. burhodogranariea B* with 8 µM CecA, (B) *P. burhodogranariea D* with 16 µM CecA, (C) *P. burhodogranariea B* with 4 µM CecAV, and (D) *P. burhodogranariea D* with 2 µM CecAV.
